# Neuroprotective Activity of Polyphenol-Rich *Ribes diacanthum* Pall against Oxidative Stress in Glutamate-Stimulated HT-22 Cells and a Scopolamine-Induced Amnesia Animal Model

**DOI:** 10.3390/antiox9090895

**Published:** 2020-09-21

**Authors:** Hyun Jeong Kim, Seung Yeon Baek, Dai-Eun Sok, Kun Jong Lee, Young-Jun Kim, Mee Ree Kim

**Affiliations:** 1 Department of Food and Nutrition, Chungnam National University, Daejeon 34134, Korea; 113kimhj@naver.com (H.J.K.); qor7683@o.cnu.ac.kr (S.Y.B.); 2 College of Pharmacy, Chungnam National University, Daejeon 34134, Korea; daesok@cnu.ac.kr; 3 Department of Food and Nutrition, Soongeui Women’s College, Seoul 04628, Korea; kunjong@hanmail.net; 4 Department of Food Science and Technology, Seoul National University of Science and technology, Seoul 01811, Korea; kimyj@seoultech.ac.kr

**Keywords:** *Ribes diacanthum* Pall, neuroprotective effect, Akt/Nrf2/ARE pathway, BDNF/TrkB pathway

## Abstract

*Ribes diacanthum* Pall, a native Mongolian medicinal plant, has been reported to show antioxidant activities due to its polyphenol and flavonoid content, and is especially rich in the ethyl acetate fraction from an 80% methanol extraction (RDP). We assessed the cytoprotective effect of RDP on glutamate-caused oxidative stress and apoptosis in mouse hippocampal neuronal cells (HT-22 cells). Cell viability was significantly recovered by RDP treatment. Also, RDP effectively decreased the glutamate-induced production of intracellular reactive oxygen species (ROS). In flow cytometric analysis, apoptotic cells and the mitochondrial membrane potential were suppressed by RDP. In the Western blotting analysis, we found that RDP not only decreased the release of apoptotic proteins but also recovered anti-apoptotic protein. Additionally, RDP enhanced the antioxidant defense system by regulating the expression of antioxidant enzymes. Furthermore, treatment with RDP activated the BDNF/TrkB pathway. In accordance with the in vitro results, RDP meliorated memory deficit by defending hippocampal neuronal cells against oxidative damage in scopolamine-injected mice. Taken together, our present study showed that RDP exerted antioxidant and neuroprotective actions against oxidative stress. Therefore, RDP might facilitate the development of candidates for functional health foods for neurodegenerative disorders.

## 1. Introduction

Neurodegenerative disorders, like Alzheimer’s disease (AD), Parkinson’s disease (PD), Huntington’s disease (HD), amyotrophic lateral sclerosis (ALS), and prion diseases, are characterized by the fatal loss of neuronal cells, accompanying functional regression of the nervous system in the brain [1,2]. Oxidative stress has been reported to be the main factor in progress of these neurodegenerative disorders [3]. Many associations between neuropathological processes and oxidative stress lead to neuronal damage and death [4]. In neuronal cells, oxidative stress causes the accumulation of polyglutamate aggregates, protein misfolding, changes in intracellular mechanisms, membrane damage, mitochondrial dysfunction, and programmed cell death such as apoptosis and autophagy [5,6]. In fact, comparing the brain to other organs, it is more responsive to oxidative stress for several reasons, as it contains its plentiful oxidized unsaturated fatty acids and lipid peroxidation content, consumes high oxygen per unit weight, and needs ample content of key ingredients such as iron and ascorbate to prevent the scarcity of antioxidant defense systems [7,8].

Although various mechanisms suppress oxidative stress in the brain, many previous studies have reported momentous roles for the nuclear factor E2-related factor 2 (Nrf2)- signaling pathway and the expression of brain-derived neurotrophic factor (BDNF) in neuroprotective action [9,10,11,12]. The Nrf2, as a transcription factor, regulates the expression of the antioxidant enzymes including NAD(P)H) quinone oxidoreductase 1 (NQO-1), glutamate-cysteine ligase catalytic subunit (GCLC), heme oxygenase 1 (HO-1), and glutathione (GSH)—cytoprotective enzymes that function as the first line of defense, helping cells to protect critical macromolecules such as DNA and proteins against lipid-mediated damage and oxidative stress [2,12,13,14].

Another mechanism performing neuroprotective action is related to BDNF, which regulates neuronal survival, development, and function [15,16,17,18]. BDNF, a neurotrophin, influences the proliferation, differentiation, and growth of neurons during progression of survival, but also plays a leading role survival and function of mature neurons in mammals [17,18,19,20]. BDNF exerts its biological function through binding to its receptor, tyrosine receptor kinase B (TrkB), which initiates multiple signaling cascades [10,11,12,19,20]. The cAMP-calcium response element-binding protein (CREB), which a transcription factor, is phosphorylated and activated by BDNF, mediating the indispensable transcription of genes for the survival and differentiation of neurons, [21,22]. Mature BDNF activates tyrosine receptor kinase B (TrkB) signaling, and then TrkB activates the downstream effectors, including protein kinase B (Akt) and extracellular signal-regulated kinase (ERK) [10,21].

In recent years, there has been a growing interest in the beneficial effects of some antioxidant substances contained in commonly used traditional native plants in preventing neurodegenerative diseases [10,11,12,23,24,25]. *Ribes diacanthum* Pall (Mongolian: Thehiin Sheeg), a native Mongolian medicinal plant, is a sort of the Saxifragaceae family that is spread throughout the northern Mongolian regions including Khangai, Khentii, Great Khingan, Mongol Daguur, and East Mongolia [26]. Its parts (leaves, stem, and fruit) have been used as a traditional medicine in the treatment of several disorders, such as kidney disease, cystitis, and bladder infection. Specifically, a water extract of it, as a type of tea, is a known folk medicine used for the treatment of edema and detoxification [27]. Regarding the nutrient composition of this plant, dried it is rich in nutritional components such as vitamin C, vitamin B1, vitamin B2, vitamin PP, vitamin A, vitamin E, carotene, 10 kinds of mineral elements, and 18 kinds of amino acid, and is of high edible value [28]. Previously, we found that the ethyl acetate extract of *Ribes diacanthum* Pall (RDP) contained high amounts of total phenols and flavonoids [26]. Additionally, we previously investigated the preventive effect of RDP on high blood glucose levels in alloxan-induced diabetic mice [29]. Furthermore, in a recent study, we reported the antioxidant and anti-inflammatory effects of RDP in both lipopolysaccharide (LPS)-treated RAW 264.7 cells and the 12-O-tetradecanoylphorbol-13-acetate (TPA)-induced dermatitis mice model, and its potential molecular mechanisms [30]. Nonetheless, the neurodegenerative effect of RDP has not been investigated and elucidated.

Here, we examined whether RDP could protect hippocampal neuronal cells against oxidative damage-induced apoptosis and cell death in both experiments in vitro and in vivo. This present study proposes the neuroprotective effect of RDP related to BDNF and effects on the antioxidant defense system by activation of the TrkB pathway. Moreover, such an effect of RDP can support basic knowledge for the practical use of RDP as a preventative supplement for neurodegenerative disorders.

## 2. Materials and Methods

### 2.1. Chemicals

Dulbecco’s modified Eagle’s medium (DMEM), 10 × phosphate-buffered saline (10 × PBS), 10 × Tris-buffered saline (10 × TBS), and Hank’s balanced salt solution (HBSS) were purchased from Welgene, Inc. (Deague, Korea). Fetal bovine serum (FBS), 2′, 7′-dichlorodihydroflorescein diacetate (DCF-DA), and 2′, 7′-dichlorofluorescein (DCF) were purchased from Invitrogen, Inc. (Carlsbad, CA, USA). Trypsin-EDTA (ethylenediaminetetraacetic acid) and antibiotics (penicillin and streptomycin) were obtained from Gibco, Inc. (BRL Life Technologies, Grand Island, NY, USA). The EZ-Cytox Cell Viability Assay Kit (CYT3000) was purchased from LPS Solution, Inc. (Seoul, Korea). PRO-PREP protein extraction solution was purchased from Intron, Inc. (Gyeonggi-do, Korea). The Nuclear Extraction Kit (10009277-1) was purchased from Cayman Chemical (Ann Arbor, MI, USA). The GSH assay kit (EGTT-100) was purchased from Bioassay Systems (Hayward, CA, USA). Sodium phosphate-monobasic, sodium phosphate-dibasic, acetic acid, potassium phosphate-monobasic, and potassium phosphate-dibasic were purchased from Daejung, Ltd. (Chung-Won, Korea). 4,6-dihydroxy-2-mercaptopyrimidine was purchased from Alfa Aesar (Seoul, Korea). Ethylenediaminetetraacetic acid (EDTA) was purchased from Junsei Chemical Co, Ltd. (Chuo-ku, Tokyo). The Muse Annexin V and Dead Cell Kit (MCH100105), Muse Mitopotential Kit (MCH100110) was purchased from Sigma Aldrich, Inc. (St. Louis, MO, USA). L-glutamic acid, dimethyl sulfoxide (DMSO), sodium dodecyl sulfate (SDS), K252a, β-nicotinamide adenine dinucleotide 2′-phosphate reduced tetrasodium salt hydrate (NADPH), 4,6-dihydroxy-2-mercaptopyrimidine (DTNB), glutathione reductase (GR, type Ⅲ from baker’s yeast), L-glutathione reduced (GSH), and TWEEN-20 were purchased from Sigma Aldrich, Inc. (USA). Anti-GCLC (ab53179, 1:2000), anti-NQO-1 (ab34173, 1:2000), and anti-cytochrome C (ab90529, 1:1000) antibodies were obtained from Abcam, Inc. (Cambridge, UK). HO-1 (ADI-OSA-110-D, 1:1000) antibodies were purchased from Enzo Life Sciences, Inc. (Farmingdale, NY, USA). Antibodies, including Bcl-2 (#3498, 1:1000), apoptosis-inducing factor (AIF, #4642, 1:1000), Bax (#2772, 1:1000), calpain, Nrf2 (#12721, 1:1000), phosphor-TrkB (#4619, 1:1000), phospho-CREB (#9198, 1:500), β-actin (#4970, 1:2000), and anti-rabbit IgG, were brought from Cell Signaling Technology, Inc, (Danvers, MA, USA). BDNF (sc-546, 1:1000) was purchased from Santa Cruz Biotechnology, Inc. (Dallas, TX, USA). Polyvinylidene difluoride (PVDF) membranes were purchased from Bio-Rad Laboratories (Hercules, CA, USA). The enhanced chemiluminescent (ECL) detection reagents were purchased from Intron, Inc. (Gyeonggi-do, Korea). X-ray film was obtained from AGFA, Inc. (ON, Canada).

### 2.2. Preparation of Ribes diacanthum Pall (RDP) Extract

**Figure 1:**
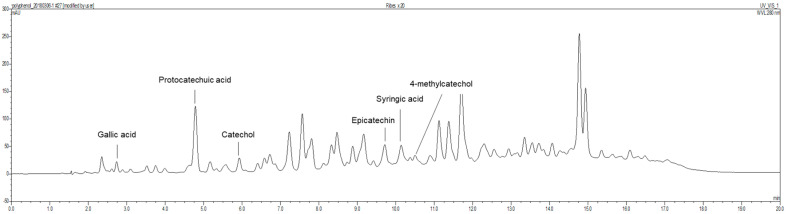
Chromatographic profile obtained with HPLC in ethyl acetate extract of *Ribes diacanthum* Pall.

*Ribes diacanthum* Pall was obtained from Mongolia in the Khentii Mountains located in the northern regions of Mongolia. RDP was prepared according to a method in a previous report [30]. Briefly, the dried and lyophilized *Ribes diacanthum* Pall (3 kg) was extracted with 6 L of 80% methanol in a sonicator bath for 3 days. The extract with 80% methanol was filtered by Whatman No. 2 filter paper (Whatman International Limited, Kent, UK). The above process was repeated 3 times. The whole mixture by filtering was concentrated using a rotary evaporator (N11, Yamato Co., Tokyo, Japan). The concentrate was dissolved in distilled water, added to ethyl acetate (1:1, *v*/*v*), and partitioned in a separatory funnel. The fraction was evaporated and concentrated in a rotary evaporator at temperatures under 40 °C (yield, 41 g). The total phenolic compounds and flavonoid contents in RDP were 0.488 mg/mL and 0.995 mg/mL, respectively [26]. In a previous report, as shown in Figure 1, we showed that RDP contained six polyphenolic substances, including gallic acid (6.28 mg/100 g) at 2.731 min, protocatechuic acid (121.96 mg/100 g) at 4.783 min, catechol (30.86 mg/100 g) at 5.926 min, epicatechin (92.31 mg/100 g) at 9.711 min, syringic acid (19.14 mg/100 g) at 10.137 min, and 4-methyl catechol (4.55 mg/100 g) at 10.731 min, respectively, by performing HPLC with each standard [30]. The extract was diluted 20-times with diluted solution (KH_2_PO^4^:MeOH:D.W = 2:3:15), and the diluted solution was filtered b a 0.45 μm syringe filter. The filtered samples were analyzed using HPLC (Thermo Fisher UltiMate 3000, Thermo Scientific, Bremen, Germany), and Eclipse XDB-C18 column (150 × 4.6 mm, 5 μm, Agilent Technologies, Wilmington, DE, USA) at 40 °C. 3% Acetic acid was used as the HPLC mobile phase, and the detector was monitored to a wavelength of 280 nm and a flow rate of 1.0 mL/min, where 10 μL of the samples were injected and analyzed. The standard calibration curve was measured by the following as standards: gallic acid, protocatechuic acid, catechol, catechin, chlorogenic acid, epigallocatechin gallate, caffeic acid, epicatechin, syringic acid, 4-methycatechol, epicatechin gallate, p-coumaric acid, ferulic acid, and rutin. The content was expressed as mg/100 g FW. The sample was stored at −20 °C until used.

### 2.3. Cell Culture

The HT-22 cells, mouse hippocampus-derived neuronal cell line, were obtained from Medifron (Seoul, Korea). The HT-22 cells, whose passage number were from 4 to 12, incubated in Dulbecco’s modified Eagle’s medium (DMEM) with 10% FBS, 100 units/mL penicillin and 100 μg/mL streptomycin at 37 °C with 5% CO_2_. All the in vitro experiment contains a vehicle control group dissolved with 0.1% DMSO.

### 2.4. Measurement of Cell Viability

Cell viability in HT-22 cells was investigated according to a previously reported method [10]. Briefly, the HT-22 cells were seeded into 96-well plates at a density of 3 × 10^3^ cells/well for 24 h, with each well containing 100 μL of medium. The cells were treated with glutamate (5 mM) in the presence or absence of RDP (5–50 μg/mL). After 11 h of incubation, cell viability was evaluated using the EZ-Cytox Cell Viability Assay Kit following the manufacturer’s instructions. The absorbance was measured by an Emax Precision microplate reader (Molecular Devices, Sunnyvale, CA, USA) at 450 nm. The percentage of surviving cells was calculated relative to the values of vehicle control group.

### 2.5. Measurement of Intracellular ROS Levels

The measurement of intracellular reactive oxygen species (ROS) levels was examined by using 2′,7′-dichlorofluorescein diacetate (DCFDA) as following our previous report [10]. Prior to glutamate treatment, the HT-22 cells were stained and incubated with 10 μM DCFDA in Hank’s balance salt solution (HBSS) for 30–60 min in the darkness, and the fluorescence was estimated by using a microplate reader (Beckman Coulter DTX 880 Multimode Detector, Brea, CA, USA) at an excitation wavelength of 485 nm and an emission wavelength of 525 nm.

### 2.6. Cell Death Assessment by Flow Cytometry

Apoptotic and necrotic cells and mitochondria membrane potential (MMP) were analyzed by flow cytometry using the Muse Annexin V and Dead Cell Kit and the Muse Mitopotential Kit, respectively. HT-22 cells were seeded on 60 mm plates and grown for 24 h. The seeded cells were treated with each concentrations of RDP (0–50 μg/mL) for 12 h, with or without 5 mM glutamate. Both the floating and attached cells were harvested and washed twice with PBS for apoptosis analysis. The measurement of the methods were followed by a manufacturer’s instruction and a previous report [10]. Approximately 10,000 cells for each group were measured and calculated the percentage of apoptotic and necrotic cells was calculated in experiments performed in triplicate by statistical analysis of the various dot plots using Muse 1.1.2 analysis software (Sigma-Aldrich, St. Louis, MO, USA).

### 2.7. Measurement of Total GSH (Glutathione) Content

The intracellular glutathione concentrations were measured using a GSH assay kit according to the manufacturer’s protocol. The cell lysate was prepared as follows. Cells (3.0 × 10^5^ in 60 mm × 15 mm plates) were washed and collected in 1 mL of ice-cold PBS and centrifuged (3000 rpm, 5 min). The cell pellet was resuspended by sonication in 200 μL of cold lysis buffer (1× PBS containing 50 mM phosphate (pH 6–7) and 1 mM EDTA) and then centrifuged at 10,000 rpm for 15 min at 4 °C. The supernatant was mixed with kit Reagent A and centrifuged (14,000 rpm, 5 min). Then, the cells were transferred to a fresh microplate and mixed with Reagent B. After incubation for twenty-five min at room temperature, the absorbance was measured at 412 nm in a microplate reader. The GSH concentration in the sample was calculated by the following formula:

Glutathione (μM) = (OD_sample_**−** OD_Blank_)/(OD_Calibrator_ − OD_Blank_) × 100 × dilution factor (μM)

### 2.8. Nuclear and Cytosolic Protein Extraction

Nuclear and cytosolic proteins were separated in RDP-treated HT-22 cells using a Nuclear Extraction Kit according to the manufacturer’s instructions (Cyaman Chemical, Ann Arbor, MI, USA).

### 2.9. Western Blot Analyses

Westernblot analysis was examined following a previous method [10]. Briefly, the total cellular proteins were fractionated by using PRO-PREP Protein Extraction solution (iNtRON Biotechnology, Gyeonggi-do, Korea). The proteins (20–50 μg) from the supernatant were resolved by SDS-polyacrylamide gel electrophoresis (SDS-PAGE) and transferred onto a PVDF membrane. The membrane was blocked with TBS buffer (20 mM Tris-HCl and 150 mM NaCl, pH 7.4) containing 5% BSA of biotechnical level for 2 h, and then incubated with different primary antibodies (NQO-1, GCLC, HO-1, p-ERK, p-Akt, Nrf2, AIF, Bax, calpain, Bcl-2, BDNF, p-CREB, and p-TrkB). PVDF membranes blotted proteins were visualized by using the West One (TM) Western Blot Detection System (iNtRON Biotechnology). The relative intensity of the protein expression was quantitated by densitometry (Image J, National Institutes of Health, Bethesda, MD, USA).

### 2.10. Animals

**Figure 2:**
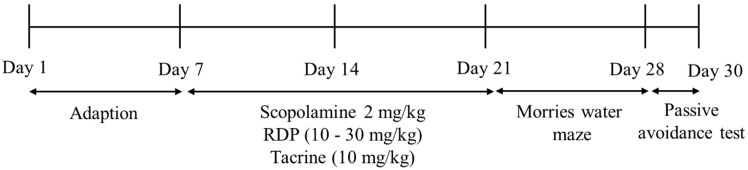
The experimental schedule.

ICR mice [9,10,12] were obtained from Raonbio, Co. (Daejeon, Korea). They are male and six-weeks-old. We exhibited the experimental schedule in Figure 2. The mice were adjusted in cages (six mice per cage) at constant temperature (25.0 ± 2.0 °C) and humidity (55 ± 10%), with a 12:12 h light/dark cycle. The mice were given free access to standard rodent food (Orientbio Inc., Seongnam, Korea) and water and weighed once a week. All animal experiments were approved by the Animal Experimental Care Center of Chungnam National University (Daejeon, Korea). The animal experiments were examined in compliance with the guidelines in the National Institutes of Health Guide for the Care and Use of Laboratory Animals (CNU-00180).

### 2.11. Morris Water Maze Test

The Morris water maze test was carried out to investigate changes of learning and memory ability from RDP treatment. A circular brown pool (1.5 m depth and 0.6 m diameter) was used by following previously conducted study [24,31]. The pool was filled with water at 23 ± 1 °C and split into quadrants. The Morris water maze test was conducted on nine days before the end of the experiment. 1 h before the behavior test, the mice orally were administered with RDP (0–30 mg/kg b.w.) or tacrine (10 mg/kg b.w.). After the administration of RDP, the mice were injected with scopolamine (2 mg/kg, i.p.).

### 2.12. Passive Avoidance Test

To investigate the cognitive ability in mice, as previously reported [18,32], passive avoidance was conducted in a light and dark chamber (Jung Bio & Plant Co. Ltd., Seoul, Korea). RDP and tacrine were administered orally, then scopolamine was injected 1 h later. After 30 min, the passive avoidance test was examined.

### 2.13. Statistical Analyses

The results are expressed as the mean ± S.E.M. All statistical analyses were performed using SPSS 24.0 (Statistical Package for Social, SPSS Inc., Chicago, IL, USA) software. We used one-way analysis of variance (ANOVA). The least significant difference (LSD) test and Duncan’s test for comparison were applied for significance between the groups. The differences among groups *p* < 0.05 and *p* < 0.01 were considered statistically significant.

## 3. Results

### 3.1. Neuroprotective Effect of RDP on Glutamate-Induced Oxidative Stress in HT-22 Cells

**Figure 3:**
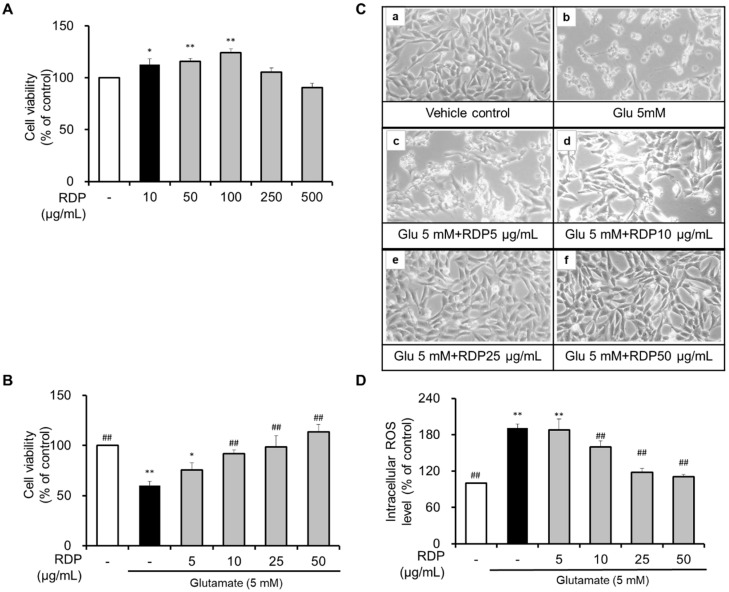
Neuroprotective effect of ethyl acetate extract of Ribes diacanthum Pall (RDP) on glutamate-induced cytotoxicity in HT-22 cells. (**A)** and (**B)** Cell viability, (**C**) morphology change in (**a**) vehicle control and cells treated with (**b**) glutamate 5 mM; (**c**) glutamate 5 mM + RDP 5 μg/mL; (**d**) glutamate 5 mM + RDP 10 μg/mL; (**e**) glutamate 5 mM + RDP 25 μg/mL, and (**f**) glutamate 5 mM + RDP 50 μg/mL. (**D**) Intracellular ROS levels. HT-22 cells were seeded at 3 × 10^3^ cell/well for 24 h. Then, the cells were treated with glutamate in the presence or absence of varying concentrations (0–50 μg/mL) of RDP for 12 h. Cell viability was measured using the EZ-Cytox Cell Viability Assay Kit. All data are presented as the mean ± S.E.M. The results were calculated as a percentage of the values obtained for control cells. * *p* < 0.05, and ** *p* < 0.01 versus vehicle control; ## *p* < 0.01 versus glutamate-treated group–indicates absence.

First, we investigated the cytotoxicity of RDP. As shown in Figure 3A, treatment with RDP significantly increased cell viability. Furthermore, it seemed that RDP might play a role in the growth of HT-22 cells. However, cell viability was slightly decreased by RDP treatments above 250 μg/mL. To investigate the neuroprotective effect of RDP, HT-22 cells were treated with 5–50 μg/mL RDP for 0.5 h. After treatment with RDP, the cells were exposed to glutamate (5 mM) for 12 h with or without RDP treatment. As shown in Figure 3B, compared to the vehicle control, cell viability was reduced by about 51% in the glutamate-alone-treated group (*p* < 0.05). Treatment with RDP dose-dependently increased cell viability. At RDP concentrations above 25 μg/mL, the cell viability recovered to the same level as in the vehicle control group. Changes in cell morphology were observed by microscopy (Figure 3B). Compared to the vehicle control, the glutamate-alone-treated cells showed extensive damage and did not stretch their branches (Figure 3C-b). However, when the cells are treated with RDP, the cells maintained their structure and appeared unharmed, dose-dependently. As shown in Figure 3D, treatment with glutamate increased the intracellular ROS levels in HT-22 cells. In contrast, RDP dose-dependently diminished the intracellular ROS levels (Figure 3D). These results showed that RDP had a neuroprotective effect against glutamate-induced oxidative stress in HT-22 cells.

### 3.2. Preventative Effect of RDP on Apoptosis in HT-22 Cells by Flow Cytometry

**Figure 4:**
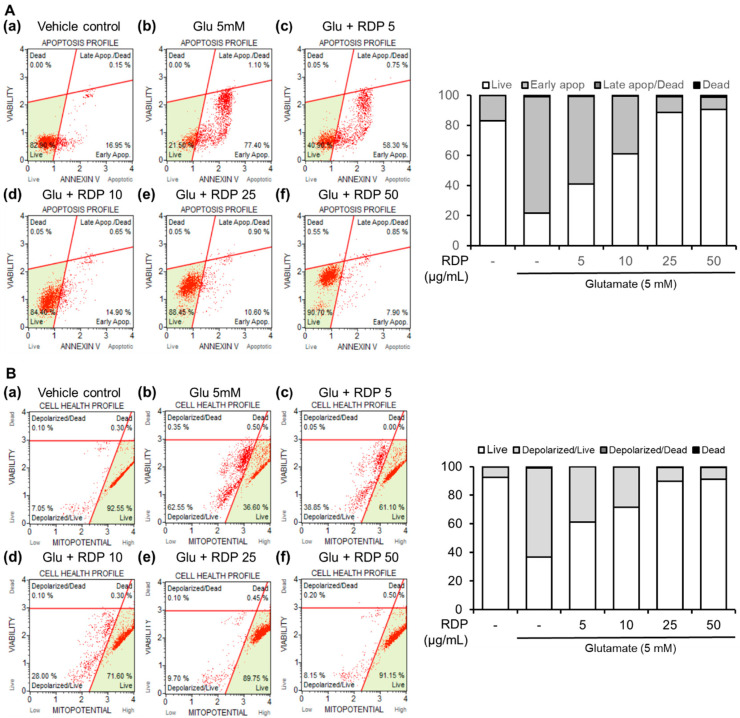
Effect of ethyl acetate extract of Ribes diacanthum Pall (RDP) on apoptotic bodies and mitochondrial membrane potential in HT-22 cells by flow cytometry. (**A**) Apoptotic bodies and (**B**) mitochondrial membrane potential in (**a**) vehicle control; (**b**) glutamate 5 mM; (**c**) glutamate 5 mM + RDP 50 μg/mL; (**d**) glutamate 5 mM + RDP 10 μg/mL; (**e**) glutamate 5 mM + RDP 25 μg/mL, and (**f**) glutamate 5 mM + RDP 5 μg/mL. HT-22 cells were seeded in 60-mm dishes for 24 h and then treated with or without RDP (0–50 µg/mL) for 30 min before glutamate challenge (5 mM). After 12 h, the apoptotic bodies and mitochondrial membrane potential in the harvested cells were analyzed by flow cytometry-indicated absence.

Next, we examined the preventative effect of RDP on oxidative stress-induced cell death in glutamate-treated HT-22 cells. ROS can react with cellular macromolecules through oxidative stress and cause the cells to undergo apoptosis [33]. As shown in Figure 4A, after treatment with glutamate alone, the HT-22 cells stained with annexin V, which detects early apoptotic cells, were noticeably increased to 77.4%, whereas 17.0% of the cells in the untreated control group were annexin V-positive. Co-treatment with glutamate and RDP markedly reduced this population of apoptotic cells. Moreover, the live cells were effectively recovered to 61.0%, 88.5, and 90.7% in HT-22 cells treated with RDP, respectively, which are dose-dependent. These results indicate that treatment with RDP inhibited oxidative stress-induced apoptotic cell death.

### 3.3. Inhibitory Effect of RDP on Mitochondrial Dysfunction in HT-22 Cells

Mitochondrial dysfunction and related glutamate-induced oxidative toxicity occur early in all major neurodegenerative diseases, and there is strong evidence that this dysfunction has a causative role in disease pathogenesis [5,33,34]. Thus, mitochondrial changes are an indicator of the health status of the cells. As shown in Figure 4B, treatment with glutamate alone reduced the MMP (live cells, 36.3%; depolarized cells, 62.55%), but co-treatment with RDP almost completely prevented the glutamate-induced MMP reduction. Moreover, the proportion of live cells was gradually increased to 91.15% by treatment with RDP at 50 μg/mL, showing recovery to the same level as that for the vehicle control group. Thus, these results demonstrate that RDP significantly inhibited mitochondrial dysfunction in glutamate-induced apoptosis.

### 3.4. Anti-Apoptotic Effect of RDP on Oxidative Stress-Induced Apoptosis

**Figure 5:**
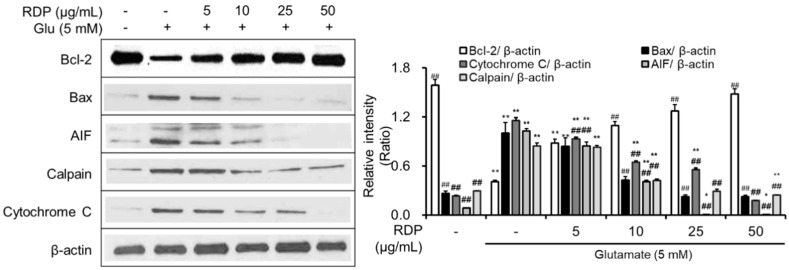
Anti-apoptotic effect of ethyl acetate extract of Ribes diacanthum Pall (RDP) on glutamate-induced apoptosis in HT-22 cells. HT-22 cells were seeded in a 60 mm dish and then incubated for 24 h. The cells were challenged with glutamate after pre-incubation with or without RDP (0–50 µg/mL) for 30 min. After twelve h, the expression of Bcl-2, Bax, AIF, calpain. cytochrome c, and β-actin was measured. The data are obtained from three independent experiments. * *p* < 0.05 and ** *p* < 0.01 versus vehicle control group. ## *p* < 0.01 versus glutamate-treated group–indicates absence.

In a previous report, we identified that elevations in ROS activated the signaling pathway related to apoptosis in hippocampus neuronal cells [9,12,17]. After we confirmed that RDP protected hippocampal neuronal cells against oxidative stress, we investigated the effect of RDP on the expression of apoptosis-related proteins in oxidative stress-exposed HT-22 neuronal cell line. RDP upregulated Bcl-2, an anti-apoptotic factor (Figure 5), whereas it downregulated the expression of pro-apoptotic factors such as Bax, AIF, calpain, and cytochrome C. These results suggest that RDP protected hippocampal neuronal cells against oxidative stress-induced apoptosis by mediating the expression of apoptosis-related proteins.

### 3.5. Activation of Antioxidant Defense System and BDNF/CREB Pathway by RDP

The activation of both the Akt/Nrf2/antioxidant response element (ARE) signaling pathway and the ERK/BDNF/TrkB pathway was reported to be related to neuroprotective effects [10,22]. Hence, we supposed that the pathways could be activated by RDP treatment because RDP protected neuronal cells against oxidative stress-induced cell death.

**Figure 6:**
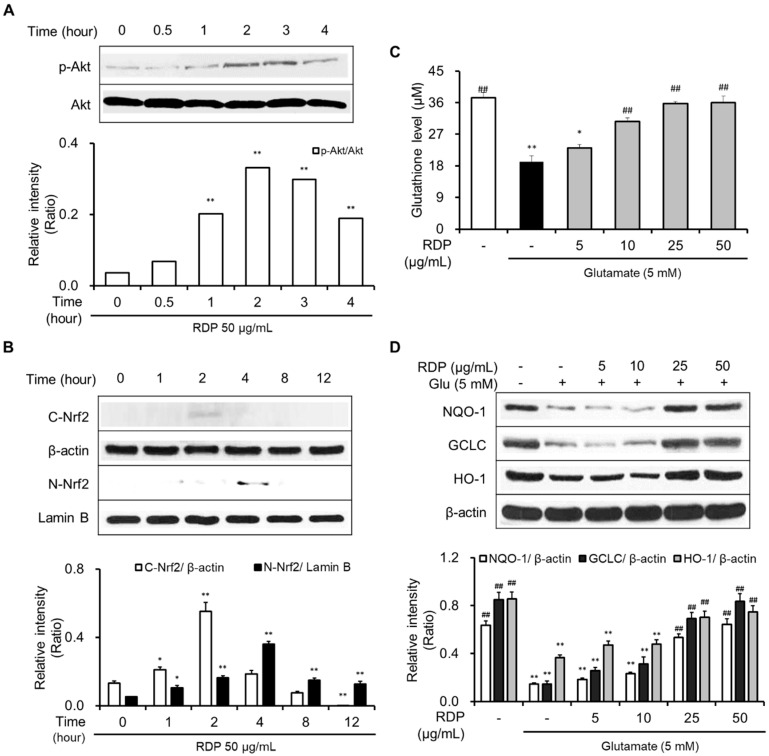
Activation of the Akt/Nrf2/ARE pathway in HT-22 cells by ethyl acetate extract of Ribes diacanthum Pall (RDP). The experiments were performed as elaborated in the Materials and Methods. The data were obtained from three independent experiments. (**A**) p-Akt, (**B**) cytosolic and nuclear Nrf2, (**C**) glutathione, and (**D**) NQO-1, GCLC, and HO-1. * *p* < 0.05, and ** *p* < 0.01 versus vehicle control group. ## *p* < 0.01 versus glutamate-treated group–indicates absence, + indicates presence.

**Figure 7:**
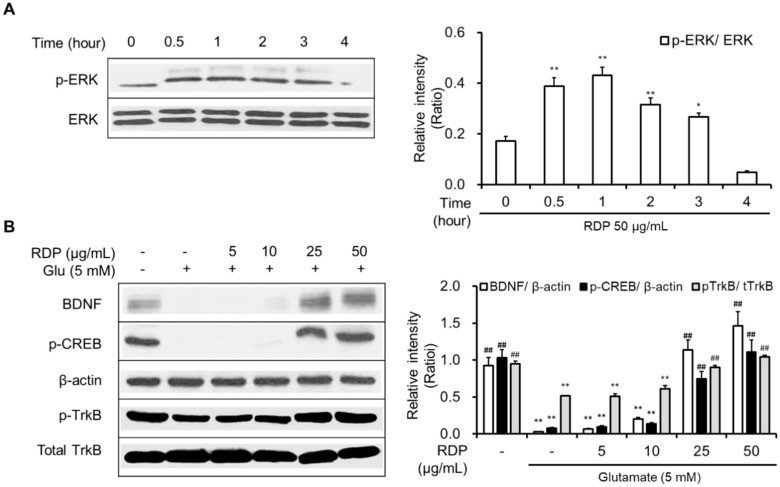
Activation of the ERK/BDNF/TrkB pathway in HT-22 cells by ethyl acetate extract of Ribes diacanthum Pall (RDP). The experiments were performed as elaborated in the Materials and Methods. The data were obtained from three independent experiments. (**A**) p-ERK, and (**B**) BDNF, p-CREB, β-actin, p-TrkB, and total TrkB. * *p* < 0.05, and ** *p* < 0.01 versus vehicle control group. ## *p* < 0.01 versus glutamate-treated group–indicates absence, + indicates presence.

As shown in Figure 6A,B, RDP activated the phosphorylation of Akt and translocated cytosolic Nrf2 to the nucleus. RDP dose-dependently enhanced the expression of GSH, NQO-1, GCLC, and HO-1 in glutamate-treated HT-22 cells (Figure 6C,D). Furthermore, RDP also promoted the expression of BDNF, p-CREB, and p-TrkB by activating p-ERK (Figure 7). Treatment with RDP at 50 μg/mL significantly recovered the expression of these proteins compared to glutamate-alone-treated HT-22 cells. These results suggest that RDP exerted neuroprotective effects via the activation of both the Akt/Nrf2/ARE and ERK/BDNF/CREB pathways.

**Figure 8:**
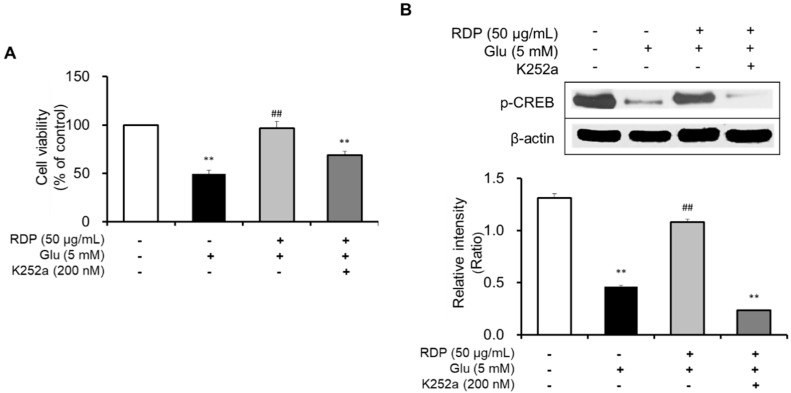
Inhibitory effect of K252a on the neuroprotective effect of ethyl acetate extract of Ribes diacanthum Pall (RDP). The experiments were performed as described in the Materials and Methods. The data were obtained from three independent experiments. (**A**) Cell viability, and (**B**) cAMP-calcium response element-binding protein (p-CREB) and β-actin. ** *p* < 0.01 versus glutamate 5 mM + RDP 50 μg/mL group. ## *p* < 0.01 versus glutamate-treated group–indicates absence, + indicates presence.

Subsequently, we clarified the mechanism by which RDP promoted activation of the ERK/BDNF/TrkB pathway in oxidative stress-induced HT-22 cells. To determine the action of RDP in the pathway, we investigated the suppressive effect of K252a, a TrkB inhibitor, in glutamate-treated HT-22 cells. After treatment with RDP, the cells were treated with K252a and pre-incubated prior to glutamate treatment. The addition of K252a significantly exacerbated the neuroprotective effects of RDP on oxidative stress-induced apoptosis in Figure 8A (*p* < 0.01). Further, the expression of p-CREB, as a transcription factor that mediates the expression of BDNF for the survival and differentiation of neuronal cells, was decreased by treatment with K252a when incubated in combination with glutamate and RDP in Figure 8B (*p* < 0.01). These results showed that RDP may affect the activation of BDNF/CREB/TrkB pathway.

### 3.6. Changes of Body Weight in RDP-Treated Mice

The effect of RDP on body weight in mice is shown in Table 1. The body weight was measured once a week during the experiment. The body weight of the mice was gradually elevated from about 21.7 g to 37.9 g on the last day of the experiment. However, there was no significance in body weight between the groups. The results indicated importance since they indicate that the administration of RDP was not cytotoxic to mice.

### 3.7. RDP Improves Scopolamine-Induced Memory Impairment in Morris Water Maze Test

**Figure 9:**
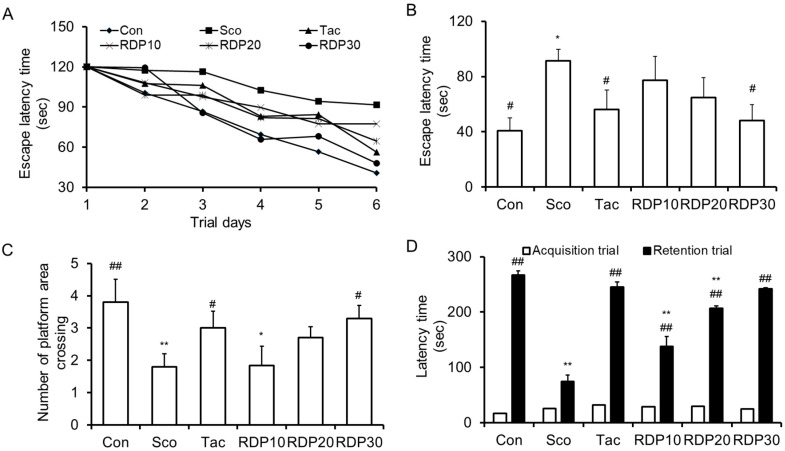
Effect of ethyl acetate extract of Ribes diacanthum Pall (RDP) on scopolamine-treated mice in behavior tests (n = 6 per group). The mice were orally administered RDP (0–30 mg/kg) before scopolamine treatment (2 mg/kg). After 1 h, the mice were tested in the Morris water maze or by the passive avoidance test (Acquisition trial). (**A**) Escape latency time during experimental days, (**B**) the number of platform area crossings, and (**C**) the latency time on the last experimental day in the Morris water maze. (**D**) Latency time in the passive avoidance test. Con: non-treated group; Sco: scopolamine 2 mg/kg-treated group; Tac: scopolamine 2 mg/kg + tacrine 10 mg/kg-treated group; RDP10: scopolamine 2 mg/kg + RDP 10 mg/kg-treated group; RDP20: scopolamine 2 mg/kg + RDP 20 mg/kg-treated group; RDP30: scopolamine 2 mg/kg + RDP 30 mg/kg-treated group. All data are the mean ± S.E.M. * *p* < 0.05 and ** *p* < 0.01 versus non-treated control group. # *p* < 0.05 and ## *p* < 0.01 versus scopolamine-treated group.

To assess the memory enhancing effect of RDP on spatial learning and memory ability in scopolamine-treated mice, the Morris water maze test was performed sequentially for six days. We observed the effect of RDP (10–30 mg/kg) on the escape latency time and the number of platform area crossings in mice, as shown in Figure 9A–C. As shown in Figure 9A,B, the escape latency time of the scopolamine-induced mice was significantly elongated than that of the vehicle control mice during all experiment periods (*p* < 0.05), implying the successful induction of memory impairment (such as amnesia) in the animal model. The administration of RDP (30 mg/kg) significantly restored the escape latency time elongated by scopolamine, similar to the results in vehicle control mice. On the last day of the experiment trial, the vehicle control mice spent about 40.8 s finding the platform, while the scopolamine-treated mice spent about 96.8 s. The escape latency time of RDP at 10, 20, and 30 mg/kg was 77.3, 64.67, and 48.0 s, respectively. The administration of RDP recovered the escape latency time extended by scopolamine. The number of platform area crossings was demonstrated by removing the platform to investigate spatial learning memory. As shown in Figure 9C, the number of platform area crossings in the scopolamine-treated mice was significantly decreased, compared to that in the vehicle control group. However, the administration of RDP (10–30 mg/kg) dose-dependently increased the number of platform area crossings. Thus, RDP may improve scopolamine-induced memory impairment in mice. Generally, many AD model studies have investigated that the effect of candidates is related with expression of antioxidant enzymes and activation of cholinergic neurotransmitters or its enzymes [9,12,18]. Considering it, RDP may affect the expression of antioxidant enzymes and cholinergic neurotransmitter, and further study is needed to find how RDP work in the oxidative stress-induced brain.

### 3.8. Enhancing Action of RDP on Memory Failure in Passive Avoidance Test

Finally, we conducted the passive avoidance test over two days just before the end of the experiment. To estimate the effect of RDP on memorizing ability, the acquisition trial and retention trial times were measured (Figure 9D). The acquisition trial time was similar between all groups. The latency time in the scopolamine-treated mice decreased significantly compared to the vehicle control group. The administration of RDP (10–30 mg/kg) exhibited a dose-dependent alleviating effect. Furthermore, the ameliorating effect of RDP (30 mg/kg) on memory deficit was the same as that of tacrine, which is an inhibitor of scopolamine and use as a treatment of neurodegenerative disease [35]. These results provide evidence that RDP can enhance the memory impairment of scopolamine-induced amnesia.

## 4. Discussion

*Ribes diacanthum* Pall has been traditionally distributed as a medicinal treatment for kidney disease, cystitis, bladder infection, and detoxification [26,27,29]. Previously, *Ribes diacanthum* Pall has been reported to contain many polyphenols and flavonoids exhibiting antioxidant activities [27]. Furthermore, *Ribes diacanthum* Pall extracted with ethyl acetate (RDP) protects against inflammatory diseases and diabetic kidney damage by downregulating oxidative stress through reductions in ROS formation by its polyphenols, which include protocatechuic acid (121.96 mg/100 g), epicatechin (92.31 mg/100 g), catechol (30.86 mg/100 g), syringic acid (19.14 mg/100 g), gallic acid (6.28 mg/100 g), and 4-methyl catechol (4.55 mg/100 g) [26,30]. Nevertheless, studies on the neuroprotective effect of RDP have not been reported yet.

Recently, we found that *Enteromorpha prolifera*, a green alga, protected hippocampal neuronal cells against oxidative stress via activation of the TrkB/Akt pathways, decreasing the expression of antioxidant enzymes and closing the BDNF autocrine loop [10,23]. Based on these reports, we supposed that RDP might inhibit oxidative stress by regulating the activation of both the Akt/Nrf2/ARE and the ERK/BDNF/TrkB pathways. Hence, we investigated the neuroprotective action of RDP on oxidative stress-induced apoptosis in HT-22 cells in vitro. First, the study found the neuroprotective effect of RDP on cell viability and the generation of ROS. Next, the effect of RDP on anti- and pro-apoptotic proteins, including Bcl-2 family, AIF, calpain, and cytochrome C was demonstrated. Finally, the study showed that RDP promoted the expression of antioxidant enzymes including GSH, NQO-1, GCLC, and HO-1 and neurotrophins including BDNF, p-CREB, and p-TrkB by activating the expression of p-Akt, p-ERK and translocating Nrf2 to the nucleus. Based on these results, an in vivo study was conducted. In the in vivo study, RDP exhibited memory improving effects in scopolamine-induced oxidative stress in mice.

As life expectancy is extended in humans, the pathogenesis of neurodegenerative disorders, such as Alzheimer’s disease (AD) in elderly people, has increased. Many studies have been reported that AD is caused by extensive oxidative stress [1,2,35]. The extensive accumulation of oxidative stress leads to cell damage and death including apoptosis and necrosis [4]. Moreover, in neuronal cells, oxidative stress causes mitochondrial dysfunction that plays a very important role in early AD [4,36]. Dysfunctional mitochondria produce less ATP needed for energy for survival and metabolism, and more reactive oxygen species (ROS) and reactive nitrogen species (RNS) [35]. Many studies have implicated metabolic defects in AD; the reduced rate of brain metabolism is one of the best-documented abnormalities in AD [36]. Then, the metabolic disorders result in the more oxidized products associated with proteins, lipids, and nucleic acid, and further alter the structure and function including impairment of synaptic plasticity, neuroinflammation, cholinergic neurotransmitter imbalance, and neuronal cell loss in the brain. As a result, oxidative stress in the brain is the crucial causative factors [37]. For this reason, extracts derived from natural plants with high antioxidant activities have been used to reduce oxidative stress. They are also preventive and therapeutic candidates for oxidative stress-induced cell death in neuronal cells because high antioxidant activity directly reduces ROS or activates the antioxidant defense system signaling pathway [5,6,7,8,10,12,24,25]. In support of this, RDP increased cell viability and decreased intracellular ROS levels in glutamate-treated HT-22 cells. In flow cytometry, RDP decreased the number of apoptotic cell bodies and reduced the MMP in glutamate-induced HT-22 cells. Glutamate is one of the major endogenous excitatory neurotransmitters in the central nervous system. Excessive amounts or high activity of glutamate can lead to neuronal disorders or even death, implicating glutamate excitotoxicity in brain diseases [38,39]. High concentrations of glutamate triggered oxidative glutamate toxicity in HT-22 cells, which lack functional ionotropic glutamate receptors [34]. The increased production of ROS following glutamate exposure was associated with increases in mitochondrial permeability changes and diffusion of the MMP and has been implicated in a variety of apoptotic phenomena [13,40]. Therefore, we suggest that RDP can protect neuronal cells against oxidative stress by mediating the generation of ROS and MMP and may exert neuroprotective effects.

One mechanism for the neuroprotective effect of RDP is associated with the generation of antioxidant enzymes such as GSH, HO-1, NQO-1, and GCLC by activating the Akt/Nrf2/ARE pathway. The nuclear factor erythroid 2-related factor 2 (Nrf2) pathway plays a significant role in cellular reductive-oxidative homeostasis, and activation of this pathway is one of the main defense mechanisms against oxidative stress [41]. In support of this, RDP treatment caused the maximum expression of phosphorylation-Akt(p-Akt) at 2-3 h and translocation of the Nrf2 into the nucleus at 4 h. The p-Akt, which is an activated form of Akt, liberates Nrf2 from the keap1-Nrf2 complex, and then Nrf2 is combined into the ARE region on DNA sequence into the nucleus after Nrf2 translocation [17]. As a result, Nrf2 promotes the expression of antioxidant enzymes in the antioxidant defense system. In support of this, RDP attributes the expression of antioxidant enzymes.

Another mechanism for the neuroprotective action of RDP, which is a crucial point in neuronal cells, is related to the upregulation of BDNF expression through activation of the ERK/TrkB/BDNF pathway. BDNF, which is a neurotrophic factor, influences cell proliferation, differentiation, and neuronal growth during development [19,20]. In the pathway, BDNF activates CREB that mediates the transcription of genes essential for the survival and differentiation in neuronal cells, and then CREB is phosphorylated [21,31]. In addition, BDNF stimulates the activation of TrkB, which is known to promote the proliferation and differentiation of neural stem cells [4,5]. Moreover, in recent studies, we found that the stimulation of TrkB was related to the activation of Akt and ERK [9,10,41]. In support of this, RDP activated the maximum expression of p-ERK and p-Akt at 1 h, or 2–3 h, respectively, in neuronal cells and then improved the expression of BDNF/TrkB/p-CREB [42]. More importantly, K252a, a TrkB inhibitor, significantly decreased the phosphorylation of CREB caused by RDP. Therefore, this suggests that RDP can exert neuroprotective effects on oxidative stress-induced neuronal cells by promoting the formation of BDNF via activation of the TrkB pathway. Consistent with the in vitro results, RDP at low doses of 10–30 mg/mL improved the performance of scopolamine-induced memory-impaired mice in the behavior test. RDP contains diverse polyphenols, including protocatechuic acid, epicatechin, catechol, syringic acid, gallic acid, and 4-methyl catechol, which have previously been reported to protect against neurodegenerative diseases [26,43,44].

Taken together, these results suggest that RDP exerts neuroprotective effects by activating both the Akt/Nrf2/ARE and ERK/TrkB/CREB pathways, which are related to the expression of antioxidant enzymes and BDNF, respectively. Therefore, RDP may possess therapeutic potential against neurodegenerative diseases. Nevertheless, further study is required to identify the active compounds in RDP that are involved in improving oxidative stress-mediated neuronal cell death and the mechanism by which RDP improves memory impairment by biochemical experiments in animal models. Moreover, more progressive studies of other AD animal models are necessary to certifying the memory improving effect of RDP for potential preventive and clinical purposes.

In summary, RDP significantly suppressed oxidative stress-induced neuronal cell death by activating the antioxidant enzyme system as well as the ERK/BDNF/CREB pathway, which may be important for the precaution and treatment of neurodegenerative disorders.

## 5. Conclusions

This study demonstrated that RDP protected against oxidative stress both in vitro and in vivo. Specifically, RDP exerted neuroprotective effects by regulating the expression of apoptotic factors, activation of the antioxidant defense system, and the ERK/BDNF/TrkB signaling pathway. The study results suggest that RDP may be a candidate for neuroprotective treatments.

## Figures and Tables

**Table 1:** Body weight of the mice.

Group		Con	Sco	Tac	RDP10	RDP20	RDP30
**Weight of Body (g)**	1 day	21.73 ± 0.08					
	7 days	29.80 ± 0.35	30.43 ± 0.35	30.03 ± 0.42	30.63 ± 0.45	29.82 ± 0.39	30.87 ± 0.44
	14 days	32.73 ± 0.50	33.00 ± 0.41	32.52 ± 0.60	33.07 ± 0.56	32.85 ± 0.62	33.40 ± 0.31
	21 days	34.78 ± 0.68	34.90 ± 0.87	34.57 ± 0.80	34.45 ± 0.58	36.15 ± 0.62	35.47 ± 0.86
	28 days	36.35 ± 0.94	35.42 ± 0.81	35.00 ± 0.77	35.60 ± 0.52	36.73 ± 0.76	36.45 ± 0.70
	31 days	36.83 ± 0.79	35.77 ± 0.71	35.17 ± 0.86	35.70 ± 0.55	36.50 ± 0.76	37.92 ± 0.84

Con: non-treated group; Sco: scopolamine 2 mg/kg-treated group; Tac: scopolamine 2 mg/kg + tacrine 10 mg/kg-treated group; RDP10: scopolamine 2 mg/kg + RDP 10 mg/kg-treated group; RDP20: scopolamine 2 mg/kg + RDP 20 mg/kg-treated group; RDP30: scopolamine 2 mg/kg + RDP 30 mg/kg-treated group. All data are the mean ± S.E.M (n = 6 per group). There was no significance between the groups.

## Abbreviations

AIF	apoptosis-inducing factor;
Akt	protein kinase B;
Bax	Bcl-2-associated X protein;
Bcl-2	B-cell lymphoma 2;
BDNF	brain-derived neurotrophic factor;
CREB	cAMP response element-binding protein;
ERK	extracellular signal-regulated kinase;
GCLC	glutamate-cysteine ligase catalytic subunit;
HO-1	heme oxygenase-1;
NQO-1	NAD(P)H quinine oxidoreductase-1;
Nrf2	NF-E2-related factor-2;
ROS	reactive oxygen species;
TrkB	Tropomyosin-related kinase receptor B

## References

[1] Ross C.A., Poirier M.A. (2004). Protein aggregation and neurodegenerative disease. Nat. Med..

[2] Yang E.J., Kim G.S., Kim J.A., Song K.S. (2013). Protective effects of onion derived quercetin on glutamate mediated hippocampal neuronal cell death. Pharmacogn. Mag..

[3] Tan M., Ouyang Y., Jin M., Chen M., Liu P., Chao X., Chen Z., Chen X., Ramassamy C., Gao Y. (2013). Downregulation of Nrf2/HO-1 pathway and activation of JNK/c-Jun pathway are involved in homocysteic acid-induced cytotoxicity in HT-22 cells. Toxicol. Lett..

[4] Gliyazova N.S., Huh E.Y., Ibeanu G.C. (2013). A novel phenoxy thiophene sulphonamide molecule protects against glutamate evoked oxidative injury in a neuronal cell model. BMC. Neurosci..

[5] Lin M.T., Beal M.F. (2006). Mitochondrial dysfunction and oxidative stress in neurodegenerative diseases. Nature.

[6] Li X., Li H., Li X.J. (2008). Intracellular degradation of misfolded proteins in polyglutamine neurodegenerative diseases. Brain Res. Rev..

[7] Calabrese V., Boyd-kimball D., Scapagnini G., Butterfield A. (2004). Nitric oxide and cellular stress response in brain aging and neurodegenerative disorders: The role of vitagenes. In Vivo.

[8] Scapagnini G., Vasto S., Abraham N.G., Caruso C., Zella D., Fabio G. (2011). Modulation of Nrf2/ARE pathway by food polyphenol: A nutritional neuroprotective strategy for cognitive and neurodegenerative disorders. Mol. Neurobiol..

[9] Lee B.D., Yoo J.M., Baek S.Y., Li F.Y., Sok D.E., Kim M.R. (2020). 3, 3′-Diindolylmethane promotes BDNF and antioxidant enzyme formation via TrkB/Akt pathway activation for neuroprotection against oxidative stress-induced apoptosis in hippocampal neuronal cells. Antioxidants.

[10] Baek S.Y., Kim M.R. (2020). Neuroprotective Effect of Carotenoid-Rich *Enteromorpha prolifera* Extract via TrkB/Akt Pathway against Oxidative Stress in Hippocampal Neuronal Cells. Mar. Drugs.

[11] Johnson J.A., Johnson D.A., Kraft A.D., Calkins M.J., Jakel R.J., Vargas M.R., Chen P.C. (2008). The Nrf2-ARE pathway: An indicator and modulator of oxidative stress in neurodegeneration. Ann. N. Y. Acad. Sci..

[12] Shin S.K., Yoo J.M., Li F.Y., Baek S.Y., Kim M.R. (2019). Mulberry fruit improves memory in scopolamine-treated mice: Role of cholinergic function, antioxidant system, and TrkB/Akt signaling. Nutr. Neurosci..

[13] Nguyen C.N., Kim H.E., Lee S.G. (2013). Caffeolyserotonin protects human keratinocyte HaCaT cells against H2O2-induced oxidative stress and apoptosis through upregulation of HO-1 expression via activation of the PI3K/Akt/Nrf2 pathway. Phytother. Res..

[14] Kim C.R., Jeon H.L., Shin S.K., Kim H.J., Ahn C.W., Jung S.U., Park S.H., Kim M.R. (2014). Neuroprotective action of deer bone extract against glutamate or Aβ 1–42-induced oxidative stress in mouse hippocampal Cells. J. Med. Food.

[15] Wang C.X., Song J.H., Song D.K., Yong V.W., Shuaib A., Hao C. (2006). Cyclin-dependent kinase-5 prevents neuronal apoptosis through ERK-mediated upregulation of Bcl-2. Cell Death Differ..

[16] Wang R., Li Y.B., Li Y.H., Xu Y., Wu H.L., Li X.J. (2008). Curcumin protects against glutamate excitotoxicity in rat cerebral cortical neurons by increasing brain-derived neurotrophic factor level and activating TrkB. Brain Res..

[17] Yoo J.M., Lee B.D., Sok D.E., Ma J.Y., Kim M.R. (2017). Neuroprotective action of n-acetyl serotonin in oxidative stress-induced apoptosis through the activation of both trkb/creb/bdnf pathway and akt/nrf2/antioxidant enzyme in neuronal cells. Redox Biol..

[18] Min A.Y., Doo C.N., Son E.J., Sung N.Y., Lee K.J., Sok D.E., Kim M.R. (2015). N-palmitoyl serotonin alleviates scopolamine-induced memory impairment via regulation of cholinergic and antioxidant systems, and expression of BDNF and p-CREB in mice. Chem. Biol. Interact..

[19] Numakawa T., Suzuki S., Kumamaru E., Adachi N., Richards M., Kunugi H. (2010). BDNF function and intracellular signaling in neurons. Histol. Histopathol..

[20] Zhang F., Kang Z., Li W., Xiao Z.-C., Zhou X.-F. (2012). Roles of brain-derived neurotrophic factor/tropomyosin-related kinase B (BDNF/TrkB) signalling in Alzheimer’s disease. J. Clin. Neurosci..

[21] Ji J.F., Ji S.J., Sun R., Li K., Zhang Y., Zhang L.Y., Tian Y. (2014). Forced running exercise attenuates hippocampal neurogenesis impairment and the neurocognitive deficits induced by whole-brain irradiation via the BDNF-mediated pathway. Biochem. Biophys. Res. Common..

[22] Yan L., Xu X., He Z., Wang S., Zhao L., Qiu J., Wang D., Gong Z., Qiu X., Huang H. (2020). Antidepressant-like effects and cognitive enhancement of coadministration of chaihu shugan san and fluoxetine: Dependent on the BDNF-ERK-CREB signaling pathway in the hippocampus and frontal cortex. BioMed Res. Int..

[23] Baek S.Y., Li F.Y., Kim D.H., Kim S.J., Kim M.R. (2020). *Enteromorpha prolifera* extract improves memory in scopolamine-treated mice via downregulating amyloid-β expression and upregulating BDNF/TrkB pathway. Antioxidants.

[24] Du C.N., Min A.Y., Kim H.J., Shin S.K., Yu H.N., Sohn E.J., Park S.H., Kim M.R. (2015). Deer bone extract prevents against scopolamine-induced memory impairment in mice. J. Med. Food.

[25] Li F.Y., Kim M.R. (2019). Effect of Aged Garlic Ethyl Acetate Extract on Oxidative Stress and Cholinergic Function of Scopolamine-Induced Cognitive Impairment in Mice. Prev. Nutr. Food Sci..

[26] Birasuren B., Oh H.L., Kim C.R., Kim N.Y., Jeon H.R., Kim M.R. (2012). Antioxidant activities of *Ribes diacanthum* Pall extracts in the northern region of mongolia. Prev. Nutr. Food Sci..

[27] Ligaa U., Davaasuren B., Ninjil N. (2006). Medicinal Plants of Mongolia Used in Western and Eastern Medicine.

[28] Khasbagan S., Xing-he G.E.N.G., Orgil J.I.N., Shan C.H.E.N. (2007). Nutritional contents of the fruits of *Ribes diacanthum* Pall. and its evaluation of edible value. J. Inner Mong. Norm. Univ..

[29] Kim E.S., Birasuren B., Kim M.R. (2013). Preventive action of *Ribes diacanthum* Pall. against high blood glucose level in alloxan-induced diabetic mice. J. East Asian Soc. Diet. Life.

[30] Jin M.C., Yoo J.M., Sok D.E., Kim M.R. (2014). Neuroprotective effect of N-acyl 5-hydroxytryptamines on glutamate-induced cytotoxicity in HT-22 cells. Neurochem. Res..

[31] Morris R. (1984). Developments of a water-maze procedure for studying spatial learning in the rat. J. Neurosci. Method.

[32] Lorenzini C.G.A., Baldi E., Bucherelli C., Sacchetti B., Tassoni G. (1997). Role of ventral hippocampus in acquisition, consolidation and retrieval of rat’s passive avoidance response memory trace. Brain Res..

[33] Loh K.P., Huang S.H., De Silva R., Tan B.K., Zhu Y.Z. (2006). Oxidative stress: Apoptosis in neuronal injury. Curr. Alzheimer Res..

[34] Fukui M., Song J.H., Choi J., Choi H.J., Zhu B.T. (2009). Mechanism of glutamate induced neurotoxicity in HT 22 mouse hippocampal cells. Eur. J. Pharmacol..

[35] Zhu X., Su B., Wang X., Smith M.A., Perry G. (2007). Causes of oxidative stress in Alzheimer disease. Cell. Mol. Life Sci..

[36] Blass J.P. (2000). The mitochondrial spiral: An adequate cause of dementia in the Alzheimers syndrome. Ann. N. Y. Acad. Sci..

[37] Gelain D.P., Antonio-Behr G., De Oliveira-Birnfeld R., Trujillo M. (2012). Antioxidant therapies for neurodegenerative diseases: Mechanisms, current trends, and perspectives. Oxid. Med. Cell. Longev..

[38] Choi D.W. (1985). Glutamate neurotoxicity in cortical cell culture is calcium dependent. Neurosci. Lett..

[39] Coyle J.T., Puttfarcken P. (1993). Oxidative stress, glutamate, and neurodegenerative disorders. Science.

[40] Pastorino J.G., Chen S.T., Tafani M., Snyder J.W., Farber J.L. (1998). The overexpression of Bax produces cell death upon induction of the mitochondrial permeability transition. J. Biol. Chem..

[41] Kumar H., Kim I.S., More S.V., Kim B.W., Choi D.K. (2014). Natural product derived pharmacological modulators of Nrf2/ARE pathway for chronic diseases. Nat. Prod. Rep..

[42] Almeida R.D., Manadas B.J., Melo C.V., Gomes J.R., Mendes C.S., Graos M.M., Carvalho R.F., Carvalho A.P., Duarte C.B. (2005). Neuroprotection by bdnf against glutamate-induced apoptotic cell death is mediated by erk and pi3-kinase pathways. Cell Death Differ..

[43] Kim N.Y., Cheong S.H., Lee K.J., Sok D.E., Kim M.R. (2020). Anti-inflammatory effects of *Ribes diacanthum* Pall mediated via regulation of Nrf2/HO-1 and NF-κB signaling pathways in LPS-stimulated RAW 264.7 macrophages and a TPA-induced dermatitis animal model. Antioxidants.

[44] Bhullar K.S., Rupasinghe H.P. (2013). Polyphenols: Multipotent therapeutic agents in neurodegenerative diseases. Oxid. Med. Cell. Longev..

